# The Influence of N-Linked Glycans on the Molecular Dynamics of the HIV-1 gp120 V3 Loop

**DOI:** 10.1371/journal.pone.0080301

**Published:** 2013-11-26

**Authors:** Natasha T. Wood, Elisa Fadda, Robert Davis, Oliver C. Grant, Joanne C. Martin, Robert J. Woods, Simon A. Travers

**Affiliations:** 1 South African National Bioinformatics Institute, South African Medical Research Council Bioinformatics Unit, University of the Western Cape, Cape Town, South Africa; 2 Department of Chemistry, National University of Ireland, Maynooth, Maynooth, Ireland; 3 Complex Carbohydrate Research Centre, University of Georgia, Athens, Georgia, United States of America; 4 School of Chemistry, National University of Ireland, Galway, Galway, Ireland; George Mason University, United States of America

## Abstract

N-linked glycans attached to specific amino acids of the gp120 envelope trimer of a HIV virion can modulate the binding affinity of gp120 to CD4, influence coreceptor tropism, and play an important role in neutralising antibody responses. Because of the challenges associated with crystallising fully glycosylated proteins, most structural investigations have focused on describing the features of a non-glycosylated HIV-1 gp120 protein. Here, we use a computational approach to determine the influence of N-linked glycans on the dynamics of the HIV-1 gp120 protein and, in particular, the V3 loop. We compare the conformational dynamics of a non-glycosylated gp120 structure to that of two glycosylated gp120 structures, one with a single, and a second with five, covalently linked high-mannose glycans. Our findings provide a clear illustration of the significant effect that N-linked glycosylation has on the temporal and spatial properties of the underlying protein structure. We find that glycans surrounding the V3 loop modulate its dynamics, conferring to the loop a marked propensity towards a more narrow conformation relative to its non-glycosylated counterpart. The conformational effect on the V3 loop provides further support for the suggestion that N-linked glycosylation plays a role in determining HIV-1 coreceptor tropism.

## Introduction

The HIV-1 envelope (Env) glycoprotein plays a critical role in the recognition, binding and entry of the virus to a new host cell [1]. Upon recognition of the host target cell, the surface subunit gp120 binds to the CD4 receptor and initiates a series of conformational changes in the gp120 trimer that enables subsequent binding to a chemokine coreceptor (CCR5 or CXCR4), fusion of the HIV and host cellular membranes, and entry into the cell. The HIV-1 envelope trimer, which consists of three gp120-gp41 heterodimers, is heavily glycosylated [2]–[5] and contains more than 75 potential N-linked glycosylation sites [6]. The glycans are covalently linked to specific asparagine residues of the envelope precursor protein gp160 by the host cell machinery and are important for the stabilisation and correct folding of gp120 [7], [8]. Studies have shown that N-linked glycans bound to the gp120 trimer form a “glycan shield” protecting the virus from neutralisation by the host immune response [9]–[16]. More recently, a number of studies have isolated broadly cross neutralising (BCN) antibodies from HIV-infected individuals, the activity of which appears to be highly dependent on the presence of glycosylation at a number of positions on the gp120 trimer, particularly at position 332 (HXB2 numbering) [17]–[20].

Glycans have also been shown to increase the binding affinity of gp120 for CD4, and the extent of glycosylation has been linked to cellular coreceptor tropism [21]–[24]. Since viral coreceptor tropism is associated with cell selection and disease progression, substantial research has been undertaken to understand the differences between viruses that preferentially use one, or both, of the common chemokine coreceptors, CCR5 and CXCR4 [25]–[30]. Several regions of gp120 may influence coreceptor usage [31]–[39], but features of the gp120 V3 loop in particular are recognised as the key factors determining viral tropism [21], [22], [35]–[44]. These factors include the net charge [21], [45], mutations at particular positions along the 35 amino acids of the V3 loop region [40], [42], [44], and the presence or absence of an N-linked glycosylation site [22], [41]–[43]. The net charge and mutation profile at specific sites of the V3 loop are commonly used in coreceptor tropism prediction tools [44], [46]–[48], however the role of the glycan profile in determining tropism is less well understood. One of the reasons for this is that the glycans of glycoproteins are challenging structures to crystallise, and are therefore often excluded prior to structural investigations. The heterogeneity in carbohydrate composition and position, as well as their flexibility, inhibits crystallisation, yet many glycoproteins are dependent on these carbohydrates for folding into their native conformations [49], [50]. Most of the currently available HIV-1 Env-related crystal structures are of a de-glycosylated gp120 monomer [51]–[53], with recent studies describing a cryo-EM structure of the unliganded trimeric Env precursor [54] as well as a partially glycosylated gp120 in complex with antibodies and CD4 [55].

Molecular dynamics (MD) simulations can be used to complement experiment-based structural studies by providing insight into spatial and temporal properties under physiological conditions [56], [57]. MD simulations are particularly suited to systems that are experimentally underdetermined or poorly resolved, such as glycans and glycoproteins [58], and can provide valuable insight into the structure-function relationship of large biomolecular systems. Two recent studies used MD simulations to describe the conformational properties of gp120 and the gp120 V3 loop [59], [60], but did not address N-linked glycosylation directly.

Yokoyama *et al.* [59] focused on the potential role of the net charge of the V3 loop, where one structure with a net charge of +7 and the other with a net charge of +3 was investigated. The MD simulations of these gp120 structures led the authors to conclude that the net charge of the V3 loop affects the structural dynamics of the entire gp120 outer domain by modulating its electrostatic potential [59]. In a separate study, MD simulations were carried out on only the V3 loop regions of crystallised structures originating from two viruses, one a CXCR4-using and the other a CCR5-using virus [60]. The conclusions drawn therefore relate to the coreceptor-specific dynamics of the V3 loop and the authors suggest that an open V3 loop conformation supports X4-tropic and a thinner conformation supports R5-tropic viruses.

Using MD simulations we can investigate the degree to which glycans affect the structural fluctuations of the gp120 protein. In this study we used molecular dynamics simulations to compare glycosylated and non-glycosylated HIV-1 gp120 structures to determine the influence glycans have on the mobility of the V3 loop of the glycoprotein.

## Methods

### Data Preparation

The crystal structure of the HIV-1 gp120 core including the V3 loop (PDB ID 2B4C [52]) was used as the starting structure for the simulations. The published protein structure was crystallised in a complex with CD4 and the X4 antibody; both of which were removed. In order to allow direct comparison of our work with that of Yokoyama *et al.* [59], who used the crystal structure 2B4C as template, Gly 25 of the V3-loop (protein residue 322 of HXB2) was mutated to arginine. The resulting G25R mutant had a net charge of +3. During the pre-processing steps, both the N-terminus (ACE; –COCH_3_) and the C-terminus (NME; –NHCH­_3_) were capped to avoid introducing charges at these positions. This non-glycosylated structure was used as control. For the glycosylated structures, an online Glycoprotein Builder (http://www.glycam.com), which is part of the GLYCAM Web-tools suite [12], was used to build and covalently link a 3D structure of the high-mannose glycan Man-9 (α-Manp-(1-2)-α-Manp-(1-6)-[α-Manp-(1-2)-α-Manp-(1-3)]-α-Manp-(1-6)-[α-Manp-(1-2)-α-Manp-(1-2)-α-Manp-(1-3)]-β-Manp-(1-4)-β-GlcpNAc-(1-4)-β-GlcpNAc, Man_9_GlcNAc_2_) to specific N-glycosylation sites. Any steric clashes between glycans or between a glycan and the protein were resolved manually by rotating the phi (Φ), psi (Ψ), chi (Χ) torsions with the UCSF Chimera package [61]. A mono-glycosylated structure was prepared with a single Man-9 glycan linked at position 295 (glycosylated^295^), which is located at the border of the V3 loop. This variant was created to determine the extent to which proximity of N-linked glycans to the V3 loop was enough to affect the structure or dynamic properties of the loop. A second glycosylated variant (glycosylated^5-glycans^) was also prepared, in which glycans were linked to five key asparagine residues (295, 332, 339, 392, and 448) surrounding the V3 loop [62]-[68]. These positions were identified in terms of their relevance to coreceptor, CD4, or neutralising antibody binding [62]-[68].

### System Preparation

We used the tLEaP module of AMBER 10 [69] with the AMBER ff99SB [70] force field for the protein, and GLYCAM06g [71] force field for the carbohydrates, to generate the coordinate and topology files for both the glycosylated and the non-glycosylated proteins. The topology files were converted for use with the glycam2gmxRB.pl Perl script, kindly provided by Dr. Marko Wehle, which enabled a mixed 1-4 non-bonded scaling scheme to be used for the protein and carbohydrate force fields, as recommended. [72]–[74]. GROMACS v4.07 [75], [76] was used for setting up the system, as well as for all simulations. The non-glycosylated structure and glycosylated^5-glycans^ structure were immersed in a cubic water box of 16 nm^3^, whereas a cubic box of 14 nm_­_^3^ was defined for the glycosylated^295^ structure (the smaller size was verified to be large enough for the protein to remain immersed throughout the simulation). This solvation gave rise to system sizes of 407880 atoms (non-glycosylated), 407934 atoms (glycosylated^5-glycans^), and 273457 atoms (glycosylated^295^). The TIP3P explicit water model [77] was used for all simulations. Chloride ions were added to neutralise each system, and all electrostatic interactions were calculated within the Particle Mesh Ewald (PME) approach [78]. Constraints on hydrogen-containing bonds were imposed with the LINCS algorithm.

### Simulations

Energy minimisation was performed on all systems for 100,000 steps using the steepest decent algorithm prior to the MD simulations. All simulations were performed at 300 K. A step-wise protocol was employed for equilibration, beginning with a simulation under constant volume (NVT) conditions for 500 ps followed by switching to constant pressure (NPT) conditions at 1 atm for a further 500 ps. During these steps the non-hydrogen atoms were restrained with a force constant of 1000 kJ/mol/nm^2^. Thereafter, the restraints were removed and the systems equilibrated for a further 500 ps. A post-equilibration trajectory of 30 ns with a time step of 1 fs was collected for each system. Three further simulations of 10 ns each for the non-glycosylated and glycosylated^5-glycans^ structures were initiated from the 30 ns trajectories. Starting structures were selected from snapshots taken at between 10 and 30 ns intervals, and these uncorrelated simulations were used to demonstrate data reproducibility.

### Analysis

In order to gain a better understanding of the differences in the conformational propensity of the glycosylated and non-glycosylated gp120 proteins, the root mean square deviation (RMSD) between the C-alpha atoms for each of the glycosylated and non-glycosylated structures, relative to their corresponding initial minimised structures was calculated using the g_rms function in GROMACS [75], [76]. Using g_rmsf we also calculated the root mean square fluctuation (RMSF) for each C-alpha atom, where the average structure generated over the 5 – 30 ns range of the of the post-equilibration trajectory, was used as reference. The distance between the centers of mass for different C-alpha atom groups was calculated with the g_dist function. The distributions of the distances between C-alpha atoms at the base of the V3-loop (including the C-alpha atoms of amino acids 296–300 and 326–331, HXB2 numbering) and at the tip of the loop (308–319, HXB2 numbering), as well as between the two sides of the V3 loop (296–313 and 314–331, HXB2 numbering) for each trajectory were determined. Apart from the distance between the commonly defined regions of the base and tip [79], we also carried out an analysis on additional regions of the base (positions 296, 297 and 331) and tip (positions 312 – 315) sections of the V3 loop, which included the two cysteine C-alpha atoms as well one neighbouring C-alpha atom at the base (three being the minimum that constitutes a group for this calculation) and the C-alpha atoms of the GPGR tip region. This allowed for the further investigation into the movement of the extremes of the V3 loop. Principal component analysis (PCA) was carried out using GROMACS [75], [76] with the g_covar and g_anaeig functions. General statistical calculations were carried out in R [80]. Visual inspections were carried out using the VMD [81] and the USCF Chimera packages [61].

## Results

**Figure 1:**
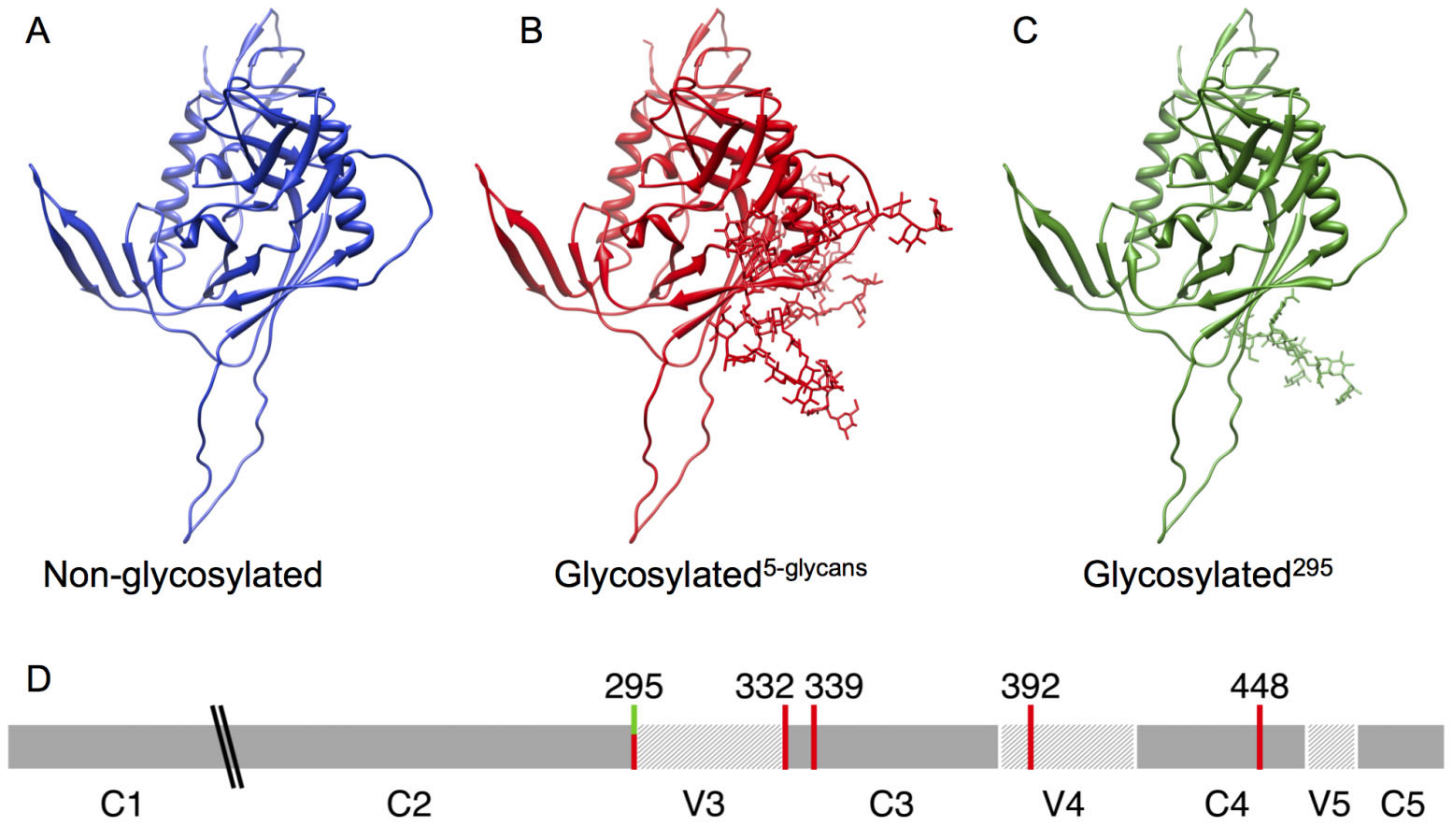
The HIV-1 gp120 (PDB ID: 2B4C) structures used in this study. These are A) non-glycosylated, B) glycosylated^5-glycans^, and C) glycosylated^295^, where the V3 loop of each structure can be seen extending out towards the bottom of each structure. D) A schematic representation of the positions (HXB2 numbering) of the investigated N-linked glycosylation sites for the glycosylated^5-glycans^ (red) and glycosylated^295^ (green) structures. The “\\” denotes the missing V1-V2 loops.

The molecular dynamic simulations we performed in this study allow us to illustrate the effect of N-linked glycosylation on the dynamics of the HIV-1 gp120 protein with specific reference to the V3 loop. A non-glycosylated structure (Figure 1A), a glycosylated structure with five glycans (glycosylated^5-glycans^, Figure 1B) and a glycosylated structure with a single glycan (glycosylated^295^, Figure 1C) were used for the comparisons. Given that the only difference between the non-glycosylated and glycosylated gp120 structures were the presence or absence of N-linked glycans bound to the surface of the protein, we expect that any conformational differences observed between the various structures are as a result of the presence or absence of the glycans.

In order to gain a better understanding of the collective dynamics of each individual structure, we plotted the RMSD values for the C-alpha atoms of all structures across each 30 ns trajectory (Figure S1). The RMSD plots illustrate the extent of movement of each structure away from the initial corresponding minimised starting structure at each time point on the trajectory. The RMSD analyses provide an indication of the overall conformational dynamics of the system and Figure S1 indicates that for each system, the conformational equilibrium for the protein backbone is reached after approximately 5 ns. The subsequent analyses were carried out on the MD trajectories from 5 to 30 ns.

**Figure 2:**
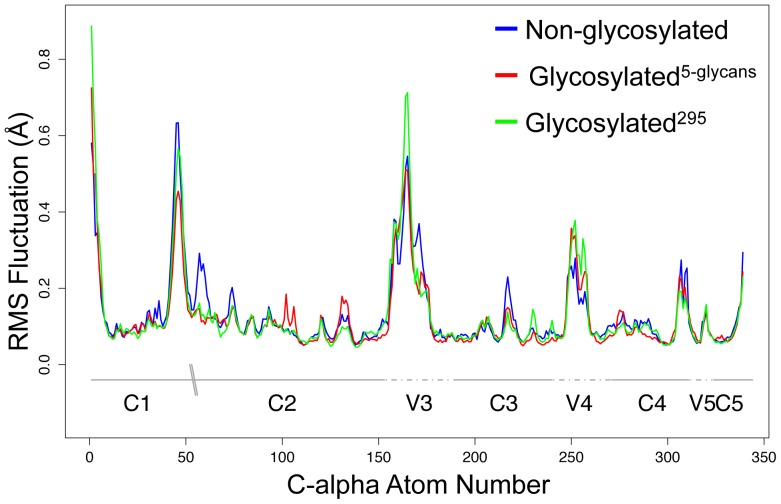
RMSF values for the C-αlpha atoms. These values represent the entire gp120 protein (based on the amino acid sequence of PDB ID: 2B4C) for the non-glycosylated, glycosylated^5-glycans^, and glycosylated^295^ trajectories. The conserved and variable regions of HIV-1 gp120 are marked, and the “\\” denotes the missing V1-V2 loops.

The RMSF profiles (Figure 2) illustrate the C-alpha atom specific fluctuations for each MD simulation (averaging over 5–30 ns for each simulation). The plots show that the loop regions are generally more dynamic than other more structured regions of the protein (Figure 2). The C-alpha atom RMSF distribution for the non-glycosylated MD simulation was significantly greater than that of the glycosylated^5-glycans^­ simulations (p < 0.05; one-sided Wilcoxon rank-sum test), indicating that glycosylation dampens the dynamics of the protein backbone. That glycosylation leads to increased fold stability has been inferred experimentally [82], [83] and predicted theoretically [83], [84], and is a generally accepted phenomena. The C-alpha RMSF distribution of the non-glycosylated simulation was also significantly greater than that of the glycosylated^295 ­^_­_system (p < 0.05; one-sided Wilcoxon rank-sum test), however no significant difference was found for the comparison between the two glycosylated, glycosylated^295^ and glycosylated^5-glycans^_­_, systems.

We also tested a further hypothesis that the effect of N-linked glycans on the protein conformational dynamics may be additive, and that the influence of a given glycan may affect only the regions surrounding the N-linked glycosylation site. In this hypothesis, the movement of the regions surrounding the glycans of the glycosylated^5-glycans^_­_ structure would be influenced more than that of the equivalent regions of the glycosylated^295^_­_ structure, but the region immediately adjacent to the N-linked glycan at position 295 will be affected in the same way for each structure. In order to test this, we compared the RMSF values for C-alpha atoms surrounding position 295 for the non-glycosylated, glycosylated^295^_­_and glycosylated^5-glycans^_­_ structures. The distribution of the C-alpha RMSF values 10 and 20 residues up- and downstream were compared using the Wilcoxon rank sum test. No significant differences were found (p > 0.05). We also compared the distribution of RMSF values for the V3 loop of all three structures and again, no significant differences were observed (p > 0.05).

**Figure 3:**
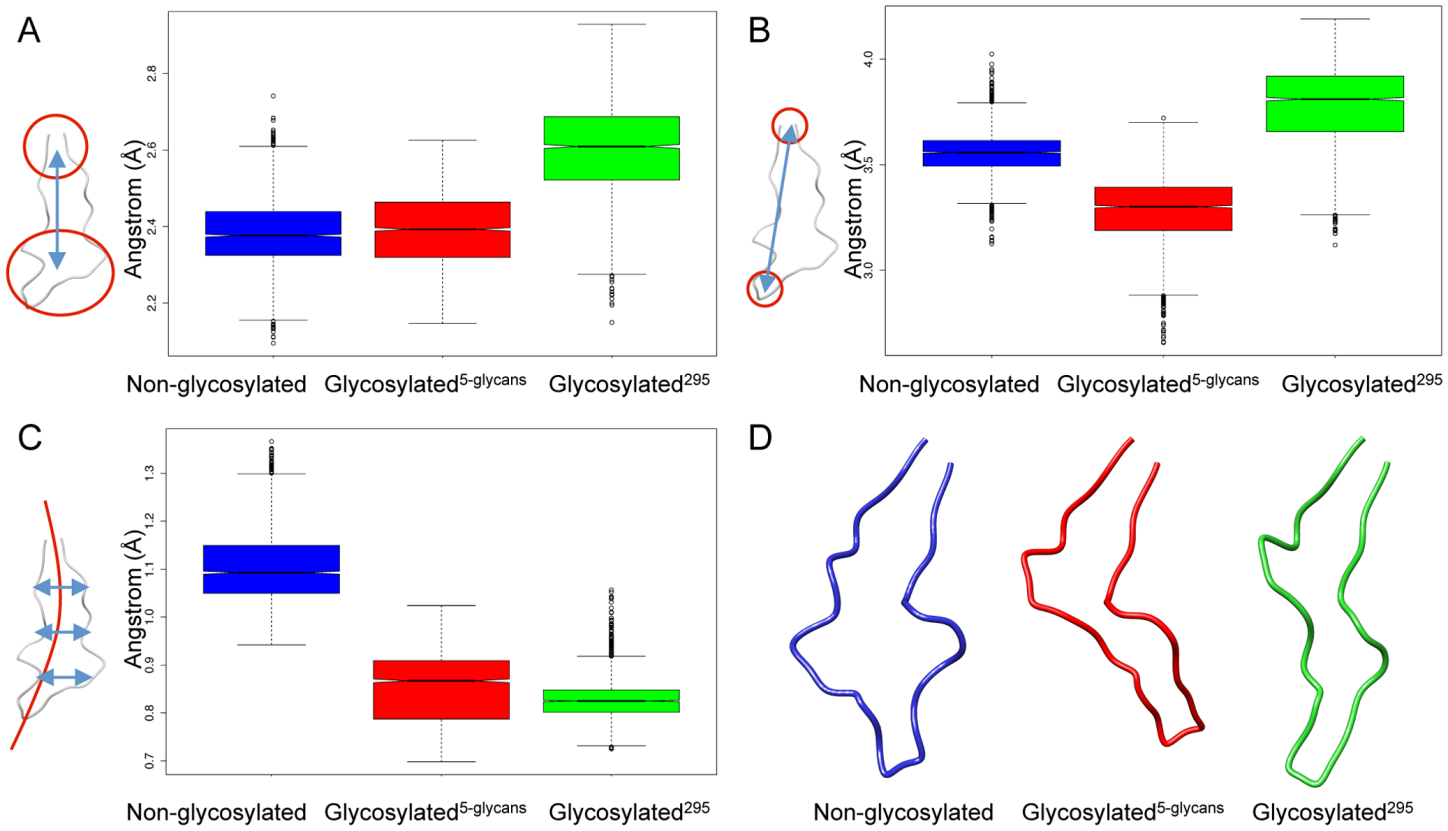
Comparison between distance distributions for regions of the V3 loop. Comparison between the distance distributions for the centers of mass between the base and tip of the V3 loop (A and B) and between the two sides of the V3 loop (C); and the corresponding average V3 loop structures (D) using 5–30 ns of the trajectories for the non-glycosylated (blue), glycosylated^5-glycans­^ (red) ­and glycosylated^295^ (green) examples.

In order to establish whether the dynamics of the V3 loop depends on the glycan profile, we calculated the distribution of the average distance between the centers of mass of the base and the tip of the V3 loop for each system (Figures 3A and 3B). In Figure 3A, the focus was on the commonly defined regions of the base (including the C-alpha atoms of amino acids 296–300 and 326–331, HXB2 numbering) and the tip (308–319, HXB2 numbering) of the V3 loop [79]. We found a significant difference (p < 0.001; Wilcoxon rank sum test) for all comparisons (Figure 3A). The average distance between the base and tip for the glycosylated^295^ V3 loop was significantly larger than for the non-glycosylated and glycosylated^5-glycans^_­_ V3 loops (Figure 3A), the latter two displaying similar distances. In the second analysis (Figure 3B) we determined the average distance between the centers of mass of the extreme groups of the V3 loop base (positions 296, 297 and 331) and tip (positions 312 – 315). Similarly, in this case we found significant differences for all comparisons of the non-glycosylated, glycosylated^295^, and glycosylated^5-glycans^_­_ structures (p < 0.001; Wilcoxon rank sum test). However, although the average distances for the glycosylated^295^ structure were still the largest, in this case the distances for the glycosylated^5-glycans^_­_ were visibly shorter than for either of the other two structures (Figure 3B). The comparisons between the distance distributions for the extreme measurements (Figure 3B) may therefore offer a more sensitive test for describing the movement of the V3 loop tip in relation to the base, but it does not reveal the direction of the movement. Since the first comparison (Figure 3A) included amino acids adjacent to the stem of the V3 loop, it is possible that the movement of the stem in relation to the base and tip influenced these measurements. The stem residues on either side of the loop may move in the same direction causing a bend in the V3 loop, or they may move in the opposite directions forming a bulge in the loop. Both these scenarios could result in a shortening between the distance of the base and tip of the V3 loop, and this effect would be more amplified when focusing on the extremes of the base and tip.

To probe variations in loop topology further, we compared the distribution of the average distance between the centers of mass of the two sides of the V3 loop (residues 296–313 and 314–331, HXB2 numbering) for all systems (Figure 3C). The difference between the distance distributions for the all the combinations of comparisons between the non-glycosylated, glycosylated^5-glycans^_­ ­_and glycosylated^295^ was highly significant (p < 0.001; Wilcoxon rank sum test; Figure 3C). However, the average side-to-side distance for the non-glycosylated loop structure was larger than seen in either of the glycosylated forms (Figure 3C). These results, together with the observations for the comparison of the distances between the base and tip, suggest that the glycosylated^295^ structure takes on a narrow elongated shape, while the glycosylated^5-glycans^_­_ takes on a narrow but bent shape (given the shorter average base-tip distance). A visual representation of the average shape of the V3 loop for each trajectory also supported this hypothesis (Figure 3D, using 5–30 ns). The V3 loop of the non-glycosylated structure had a much broader conformation with the two sides of the loop forming a bulge, whereas the glycosylated structures on the other hand showed a preference for a more narrow conformation (Figure 3D). However, the average shape does not necessarily provide a meaningful representation of the underlying dynamics of the system. Furthermore, although the distributions of distances provide an indication of the movements across the trajectories, these distances include the high frequency movements that contribute to the background noise.

**Figure 4:**
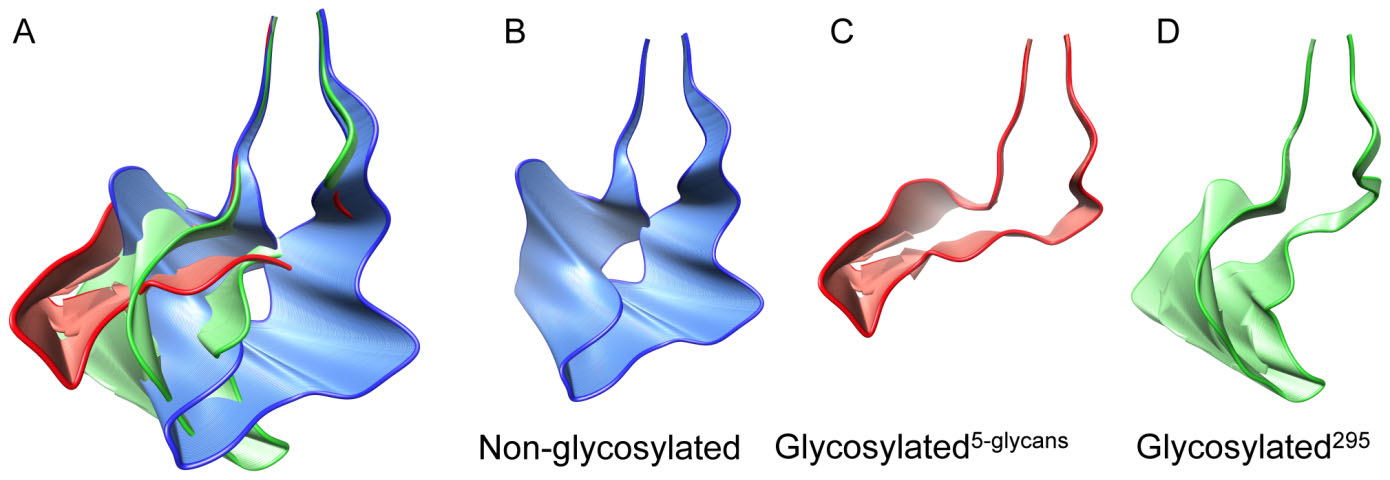
Representation of the range of movement of the V3 loops for the first principal component. The range of movement is presented for (A) all systems, (B) non-glycosylated, (C) glycosylated^5-glycans^, and (D) glycosylated^295^ trajectories. The shaded colors represent the intermediate positions between the extremes.

Principal component analysis (PCA) was carried out to describe the predominant movement of the V3 loop for each of the three MD simulations. PCA produces several principal components that describe a proportion of the system variance. For each of our structures, the first principal component (PC1) described 41%, 47% and 37% of the motion of the V3 loop for the non-glycosylated, glycosylated^5-glycans^_­ ­_and glycosylated^295^, forms respectively (Table 1). We found that the first three principal components incorporated the majority of the movement for each system, and collectively describe 63% (non-glycosylated), 73% (glycosylated^5-glycans^) and 70% (glycosylated^295^) of the total motion of each system (Table 1). In order to visualise the variance described by each principal component, we plotted the extremes, and intermediate, positions of each motion (Figure 4, and Figures S2 and S3). These representation of each of the three principal components were generally consistent with the results from the distance calculation where the V3 loop of the non-glycosylated structure takes on an open, broader, shape (Figure 4B) and the V3 loop of the glycosylated^5-glycans­^ structure has a more narrow and bent shape (Figure 4C). The V3 loop in the glycosylated^295^ structure also takes on a more narrow shape, but appears to fluctuate between elongated and bent conformations (Figure 4D). This may indicate that the additional glycans in the glycosylated^5-glycans^ form may be stabilising the bent topology.

**Table 1:** Percentage motion included in each of the first three, and the total sum of the first three, principal components (PCs).

	PC1 (%)	PC2 (%)	PC3 (%)	Total (%)
**Non-glycosylated**	41	13	9	63
**Glycosylated^5-glycans^**	47	17	9	73
**Glycosylated^295^**	37	25	8	70

In order to confirm that the results obtained from the analysis of the 30 ns trajectories were not derived from the systems populating local minima, we repeated the V3 loop analyses on three additional 10 ns simulations (started from uncorrelated snapshots) for each of the non-glycosylated and glycosylated^5-glycans^ systems. Apart from one calculation, the comparisons of the distributions of the average distance between the centers of mass of the base and the tip (both for the defined regions, Figure S4A, as well as the extremes, Figure S4B) of the V3 loop were highly significant (p < 0.001; Wilcoxon rank sum test). The comparisons between the distributions of the average distance between the centers of mass of the two sides of the V3 loop (residues 296–313 and 314–331, HXB2 numbering) for each trajectory, was highly significant (Figure S4C, p < 0.001; Wilcoxon rank sum test). These results, together with the representation of the average structures for each of the 10 ns simulations (Figure S4D), are in agreement with the initial findings (30 ns trajectory results) where the glycosylated^5-glycans^ V3 loops is more narrow than the non-glycosylated form.

The PCA of the uncorrelated snapshots MD simulations showed that the first three principal components described between 65% and 81% of the variance for each system (Table S1). The images representing the extremes, and intermediate, positions of each motion (Figures S5, S6 and S7) followed the same trend as seen with the PCA based on the 30 ns trajectories of the non-glycosylated and glycosylated^5-glycans^ structures. Indeed, the V3 loop of the non-glycosylated system favours a much broader shape (Figures S5B, S6B and S7B) compared to the glycosylated^5-glycans­^ structures, which appear to prefer narrower and bent loops (Figures S5C, S6C and S7C).

## Discussion

In this study we have investigated the impact of N-linked glycans on the dynamics of the HIV-1 gp120 protein with specific reference to the V3 loop. The dynamic properties of HIV-1 envelope proteins play an important role in the successful binding and transmission of a virion to a host cell [85], [86]. Two recent studies have used molecular dynamic simulations to describe the movement of gp120 and the gp120 V3 loop [59], [60], however the influence of glycans on the underlying movement was not explicitly addressed. Such structural studies are important to further our understanding of the dynamic interaction between HIV-1 gp120, the CD4 receptor and coreceptors. They also allow us to associate the observed dynamics with known sequence and chemical features of the V3 loop that play a role in determining coreceptor usage. These features are commonly used in coreceptor prediction tools and include specific amino acid mutations at particular positions of the V3 loop as well as the net charge [21], [22], [41]-[44], [47], [48], [87], [88]. The presence or absence of an N-linked glycosylation site within the V3 loop has also been reported to influence coreceptor tropism [22], [43], but little is known about the mode of this action. Here we used molecular dynamic simulations to describe the dynamics of the V3 loop with and without taking the potential influence of N-linked glycans into account.

Our findings suggest that the presence of N-linked glycans has a significant impact on the dynamics of the gp120 protein particularly on the movement and preferred conformation of the V3 loop. Differences in both the length of the loop, as well as its side-to-side separation, were observed as a function of glycosylation pattern. The average distance between the base and the tip of the V3 loop for all three structures provided a first look at the potential influence of a given glycan profile, and we observed a significant difference for all combinations of comparisons between the non-glycosylated, glycosylated^295^ and glycosylated^5-glycans^ structures. However, the clear difference in distances between the two sides of the V3 loop illustrated the considerable extent to which glycans may be affecting the conformation of the loop. The V3 loop of the glycosylated structures took on a significantly more narrow formation compared to that of the non-glycosylated form. The side-to-side distances together with the base-tip distances suggested that the V3 loop of the non-glycosylated structure took on an open, broader, shape, the V3 loop of the glycosylated^5-glycans^ structure was more narrow and bent, and the V3 loop of the glycosylated^295^ structure was narrow but more elongated (Figure 3D). These observations were further substantiated by the results for the additional non-glycosylated and glycosylated ^5-glycans^ examples (three uncorrelated simulations of 10ns each, Figure S4D) where the same trend was seen.

The results from the principal component analysis further show a clear difference between the coordinate space most commonly occupied by the non-glycosylated, glycosylated^295^ and glycosylated^5-glycans^ systems (Figure 4, and Figures S2 and S3). The glycans appear to constrain the breadth of movement of the V3 loop, with the tip of the loop, in particular, appearing to move actively away from the glycans. Where a single glycan adjacent to the V3 loop was present (glycosylated^295^) this observation was less distinct than for the structure with five glycans attached (glycosylated^5-glycans^). For the glycosylated^295^ structure the V3 loop fluctuated between an elongated and bent shape, and therefore the tip appeared to move away from the glycan intermittently (Figure 4D). In contrast, the V3 loop for the glycosylated^5-glycans^ structure remained in a more bent conformation with the tip moving away from the glycans consistently (Figure 4C). The significant difference observed between the RMSF distribution of the C-alpha atoms for the entire gp120 protein for the non-glycosylated and glycosylated structures (Figure 2) may suggests that the glycan surface density plays a role in the fluctuation of nearby atoms and residues. In this case, steric hindrance imposed by the N-linked glycans, or the additional weight contribution from the N-linked glycans, could affect the surrounding protein structure. If this is the case then the type and size of each glycan could further influence the fluctuation of the protein regions neighbouring N-linked glycosylation sites. However, we did not observe a significant difference between the fluctuations of the C-alpha atoms bordering the N-linked glycosylation site at position 295 for the glycosylated^295^ and the non-glycosylated forms, which may suggest that not all protein regions are as susceptible to a change in dynamics as loop regions may be. This may also reflect the relatively limited sampling of backbone conformations accessible to a 30 ns simulation. A previous study by Yokoyama *et al.* [59] also remarked that no significant change was observed in their MD simulations when a high mannose glycan was present in the V3 loop compared to when the glycan was absent, although no reference was given to the position of the added glycan. Future investigations will need to be carried out to determine whether any single glycan attached at an N-linked glycosylation site has a significant impact on the mobility of the protein.

The underlying amino acid sequences of the protein structures we used were identical and only the glycan profile differed between examples. [60]. The observation that the non-glycosylated example of the V3 loop takes on an open, broader form and the glycosylated forms thinner, elongated/bent shapes can therefore only be associated with the extent of glycosylation and not tropism. This is in contrast to a previous study where the authors used MD simulations to characterise the movement of the V3 loop regions of crystallised structures originating from two viruses, one a CXCR4-using and the other a CCR5-using virus [60]. In their study, where the V3 loop sequence for each structure was unique, the authors suggested that an open V3 loop conformation supports X4-tropic and a thinner conformation supports R5-tropic viruses [60]. We suggest, therefore, that both the underlying amino acid sequence as well as the glycosylation profile impacts the molecular dynamics of the gp120 protein and that both factors must be considered in future structural analyses.

One of the major obstacles related to HIV-1 gp120 structural investigations relates to the availability of high-resolution crystal structures that include the variable loops. Here, we have used the 2B4C gp120 crystal structure, which includes the V3 loop, to enable comparison with previous reports that used the same structure for MD simulations [59], [60]. Furthermore, previous studies have shown that the functions of the gp120 core are somewhat independent of the variable loops in that, regardless of the underlying amino acid sequence, it retains its fundamental structural characteristics and ligand binding capacity despite the absence of the variable loops [89], [90]. Therefore we expect that the core structure derived from the crystal will have a minor influence on the overall outcome of the molecular dynamic simulations relative to that of the underlying amino acid sequence and glycan composition. The loop regions of a protein are also highly dynamic [91] and therefore difficult to crystallise [92], and it is likely that the configuration of the gp120 variable loops in the crystal structure represents only one of the many possible conformations. Molecular dynamic techniques generate multiple ensembles of the variable loops and the ongoing refinement of the force field parameters will continue to improve the accuracy of the methods to describe the conformational properties of proteins [93]-[95].

A recent study describes the differences in the crystal structures of the CCR5 and CXCR4 chemokine receptors and suggests that the steric hindrances due to amino acid substitutions may present one of the major factors involved in HIV-1 coreceptor selection [96]. Given the results from our study, we hypothesise that the steric hindrances imposed by N-linked glycans may present a further determinant of coreceptor usage. While the focus of this study was not specifically to identify the structural determinants of coreceptor usage, our results suggest that future studies should focus on exploring the interplay between the underlying amino acid sequence and the glycan composition in determining coreceptor tropism.

The importance of N-linked glycosylation of gp120 is not only evident in coreceptor studies, but they also play a role in HIV-1 antibody neutralisation and escape [9]. Because the intracellular glycosylation process, which adds the glycans to proteins, is that of the host, the immune system does not recognise the viral glycans as non-self, thereby allowing the virus to escape antibody driven responses [97]. It has also been shown that N-linked glycans bound to the surface of HIV-1 gp120 form part of several important neutralising antibody epitopes and the removal of even a single glycan can completely abolish a neutralising antibody response [17], [19], [20]. Our initial glycoprotein modelling results allude to not only a protein-glycan interaction, but also a glycan-glycan interaction, in such that the movement and influence of a single glycan is affected by the presence and mobility of a second glycan at an adjacent N-linked glycosylation site. This further underlines the importance of broadening our understanding of the influence that N-linked glycans have on the various regions of HIV-1 gp120.

Our molecular dynamics results clearly illustrate the importance of taking the glycan profile of HIV-1 gp120 into account during structural investigations. Describing the temporal and spatial constraints affecting the dynamics of different regions of gp120 may provide key insight into the structural features that drive HIV evolution and allow the virus to switch between using the CXCR4 and CCR5 coreceptor for cell entry. Furthermore, these analyses will add to our understanding of how we can take advantage of these protective and adaptive characteristics to guide the design of carbohydrate-binding HIV therapies.

## Supporting Information

Figure S1 RMSF values for the C-αlpha atoms. These values represent the entire gp120 for the non-glycosylated, glycosylated^5-glycans^, and glycosylated^295^ trajectories. The shaded areas represent the part of the trajectory that was discarded as burn-in.
(TIF)

Figure S2 Representation of the range of movement of the V3 loops for the second principal component. The range of movement is presented for (A) all systems, (B) non-glycosylated, (C) glycosylated^5-glycans^, and (D) glycosylated^295^ trajectories. The shaded colors represent the intermediate positions between the extremes.
(TIF)

Figure S3 Representation of the range of movement of the V3 loops for the third principal component. The range of movement is presented for (A) all systems, (B) non-glycosylated, (C) glycosylated^5-glycans^, and (D) glycosylated^295^ trajectories. The shaded colors represent the intermediate positions between the extremes.
(TIF)

Figure S4 Comparison between distance distributions for regions of the V3 loop. Comparison between the distance distributions for the centers of mass between the base and tip of the V3 loop (A and B) and between the two sides of the V3 loop (C); and the corresponding average V3 loop structures (D) using the entire 10 ns of the uncorrelated trajectories for the three non-glycosylated (blue) and three glycosylated^5-glycans­^ (red) additional examples.
(TIF)

Figure S5 Representation of the range of movement of the V3 loops for the first principal component. The range of movement is presented for (A) non-glycosylated and glycosylated^5-glycans^, (B) non-glycosylated, and (C) glycosylated^5-glycans^ trajectories. The shaded colors represent the intermediate positions between the extremes.
(TIF)

Figure S6 Representation of the range of movement of the V3 loops for the second principal component. The range of movement is presented for (A) non-glycosylated and glycosylated^5-glycans^, (B) non-glycosylated, and (C) glycosylated^5-glycans^ trajectories. The shaded colors represent the intermediate positions between the extremes.
(TIF)

Figure S7 Representation of the range of movement of the V3 loops for the third principal component. The range of movement is presented for (A) non-glycosylated and glycosylated^5-glycans^, (B) non-glycosylated, and (C) glycosylated^5-glycans^ trajectories. The shaded colors represent the intermediate positions between the extremes.
(TIF)

Table S1 Percentage motion included in each of the first three, and the total sum of the first three, principal components (PCs) for each of the uncorrelated repeats (10 ns).
(PDF)

## References

[1] Wyatt R, Sodroski J (1998) The HIV-1 envelope glycoproteins: fusogens, antigens, and immunogens. Science 280: 1884–1888. 9632381 10.1126/science.280.5371.1884

[2] Ji X, Chen Y, Faro J, Gewurz H, Bremer J, et al (2006) Interaction of human immunodeficiency virus (HIV) glycans with lectins of the human immune system. Curr Protein Pept Sci 7: 317–324. 16918446 10.2174/138920306778017990

[3] Leonard CK, Spellman MW, Riddle L, Harris RJ, Thomas JN, et al (1990) Assignment of intrachain disulfide bonds and characterization of potential glycosylation sites of the type 1 recombinant human immunodeficiency virus envelope glycoprotein (gp120) expressed in Chinese hamster ovary cells. J Biol Chem 265: 10373–10382. 2355006

[4] Mizuochi T, Spellman MW, Larkin M, Solomon J, Basa LJ, et al (1988) Carbohydrate structures of the human-immunodeficiency-virus (HIV) recombinant envelope glycoprotein gp120 produced in Chinese-hamster ovary cells. Biochem J 254: 599–603. 2845957 10.1042/bj2540599 PMC1135120

[5] Myers G, MacInnes K, Korber B (1992) The emergence of simian/human immunodeficiency viruses. AIDS Res Hum Retroviruses 8: 373–386. 1571197 10.1089/aid.1992.8.373

[6] Korber B, Gaschen B, Yusim K, Thakallapally R, Kesmir C, et al (2001) Evolutionary and immunological implications of contemporary HIV-1 variation. Br Med Bull 58: 19–42. 11714622 10.1093/bmb/58.1.19

[7] Land A, Braakman I (2001) Folding of the human immunodeficiency virus type 1 envelope glycoprotein in the endoplasmic reticulum. Biochimie 83: 783–790. 11530211 10.1016/s0300-9084(01)01314-1

[8] Li Y, Luo L, Rasool N, Kang CY (1993) Glycosylation is necessary for the correct folding of human immunodeficiency virus gp120 in CD4 binding. J Virol 67: 584–588. 8416385 10.1128/jvi.67.1.584-588.1993 PMC237399

[9] Wei X, Decker JM, Wang S, Hui H, Kappes JC, et al (2003) Antibody neutralization and escape by HIV-1. Nature 422: 307–312. 12646921 10.1038/nature01470

[10] Ohgimoto S, Shioda T, Mori K, Nakayama EE, Hu H, et al (1998) Location-specific, unequal contribution of the N glycans in simian immunodeficiency virus gp120 to viral infectivity and removal of multiple glycans without disturbing infectivity. J Virol 72: 8365–8370. 9733886 10.1128/jvi.72.10.8365-8370.1998 PMC110215

[11] Schonning K, Jansson B, Olofsson S, Hansen JE (1996) Rapid selection for an N-linked oligosaccharide by monoclonal antibodies directed against the V3 loop of human immunodeficiency virus type 1. J Gen Virol 77 ( Pt 4): 753–758. 10.1099/0022-1317-77-4-753 8627264

[12] Bolmstedt A, Hinkula J, Rowcliffe E, Biller M, Wahren B, et al (2001) Enhanced immunogenicity of a human immunodeficiency virus type 1 env DNA vaccine by manipulating N-glycosylation signals. Effects of elimination of the V3 N306 glycan. Vaccine 20: 397–405. 11672902 10.1016/s0264-410x(01)00358-9

[13] Chackerian B, Rudensey LM, Overbaugh J (1997) Specific N-linked and O-linked glycosylation modifications in the envelope V1 domain of simian immunodeficiency virus variants that evolve in the host alter recognition by neutralizing antibodies. J Virol 71: 7719–7727. 9311856 10.1128/jvi.71.10.7719-7727.1997 PMC192123

[14] Cheng-Mayer C, Brown A, Harouse J, Luciw PA, Mayer AJ (1999) Selection for neutralization resistance of the simian/human immunodeficiency virus SHIVSF33A variant in vivo by virtue of sequence changes in the extracellular envelope glycoprotein that modify N-linked glycosylation. J Virol 73: 5294–5300. 10364275 10.1128/jvi.73.7.5294-5300.1999 PMC112584

[15] Kang SM, Quan FS, Huang C, Guo L, Ye L, et al (2005) Modified HIV envelope proteins with enhanced binding to neutralizing monoclonal antibodies. Virology 331: 20–32. 15582650 10.1016/j.virol.2004.10.005

[16] McCaffrey RA, Saunders C, Hensel M, Stamatatos L (2004) N-linked glycosylation of the V3 loop and the immunologically silent face of gp120 protects human immunodeficiency virus type 1 SF162 from neutralization by anti-gp120 and anti-gp41 antibodies. J Virol 78: 3279–3295. 15016849 10.1128/JVI.78.7.3279-3295.2004 PMC371088

[17] Walker LM, Huber M, Doores KJ, Falkowska E, Pejchal R, et al (2011) Broad neutralization coverage of HIV by multiple highly potent antibodies. Nature 477: 466–470. 21849977 10.1038/nature10373 PMC3393110

[18] Nandi A, Lavine CL, Wang P, Lipchina I, Goepfert PA, et al (2010) Epitopes for broad and potent neutralizing antibody responses during chronic infection with human immunodeficiency virus type 1. Virology 396: 339–348. 19922969 10.1016/j.virol.2009.10.044 PMC2789835

[19] Moore PL, Gray ES, Wibmer CK, Bhiman JN, Nonyane M, et al.. (2012) Evolution of an HIV glycan-dependent broadly neutralizing antibody epitope through immune escape. Nat Med. 10.1038/nm.2985 PMC3494733 23086475

[20] Pejchal R, Doores KJ, Walker LM, Khayat R, Huang P-S, et al (2011) A potent and broad neutralizing antibody recognizes and penetrates the HIV glycan shield. Science 334: 1097–1103. 21998254 10.1126/science.1213256 PMC3280215

[21] Cardozo T, Kimura T, Philpott S, Weiser B, Burger H, et al (2007) Structural basis for coreceptor selectivity by the HIV type 1 V3 loop. AIDS Res Hum Retroviruses 23: 415–426. 17411375 10.1089/aid.2006.0130

[22] Pollakis G, Kang S, Kliphuis A, Chalaby MI, Goudsmit J, et al (2001) N-linked glycosylation of the HIV type-1 gp120 envelope glycoprotein as a major determinant of CCR5 and CXCR4 coreceptor utilization. J Biol Chem 276: 13433–13441. 11278567 10.1074/jbc.M009779200

[23] Wilhelm D, Behnken HN, Meyer B (2012) Glycosylation assists binding of HIV protein gp120 to human CD4 receptor. Chembiochem 13: 524–527. 22266649 10.1002/cbic.201100740

[24] Guttman M, Kahn M, Garcia NK, Hu SL, Lee KK (2012) Solution structure, conformational dynamics, and CD4-induced activation in full-length, glycosylated, monomeric HIV gp120. J Virol 86: 8750–8764. 22674993 10.1128/JVI.07224-11 PMC3421722

[25] Zhang L, Huang Y, He T, Cao Y, Ho DD (1996) HIV-1 subtype and second-receptor use. Nature 383: 768. 8892998 10.1038/383768a0

[26] Clapham PR, McKnight A (2001) HIV-1 receptors and cell tropism. Br Med Bull 58: 43–59. 11714623 10.1093/bmb/58.1.43

[27] Goetz MB, Leduc R, Kostman JR, Labriola AM, Lie Y, et al (2009) Relationship between HIV coreceptor tropism and disease progression in persons with untreated chronic HIV infection. J Acquir Immune Defic Syndr 50: 259–266. 19194318 10.1097/QAI.0b013e3181989a8b PMC2670851

[28] Keet IP, Krijnen P, Koot M, Lange JM, Miedema F, et al (1993) Predictors of rapid progression to AIDS in HIV-1 seroconverters. AIDS 7: 51–57. 8095146 10.1097/00002030-199301000-00008

[29] Dragic T, Litwin V, Allaway GP, Martin SR, Huang Y, et al (1996) HIV-1 entry into CD4+ cells is mediated by the chemokine receptor CC-CKR-5. Nature 381: 667–673. 8649512 10.1038/381667a0

[30] Feng Y, Broder CC, Kennedy PE, Berger EA (1996) HIV-1 entry cofactor: functional cDNA cloning of a seven-transmembrane, G protein-coupled receptor. Science 272: 872–877. 8629022 10.1126/science.272.5263.872

[31] Boyd MT, Simpson GR, Cann AJ, Johnson MA, Weiss RA (1993) A single amino acid substitution in the V1 loop of human immunodeficiency virus type 1 gp120 alters cellular tropism. J Virol 67: 3649–3652. 8497073 10.1128/jvi.67.6.3649-3652.1993 PMC237718

[32] Carrillo A, Ratner L (1996) Human immunodeficiency virus type 1 tropism for T-lymphoid cell lines: role of the V3 loop and C4 envelope determinants. J Virol 70: 1301–1309. 8551600 10.1128/jvi.70.2.1301-1309.1996 PMC189948

[33] Groenink M, Fouchier RA, Broersen S, Baker CH, Koot M, et al (1993) Relation of phenotype evolution of HIV-1 to envelope V2 configuration. Science 260: 1513–1516. 8502996 10.1126/science.8502996

[34] Smyth RJ, Yi Y, Singh A, Collman RG (1998) Determinants of entry cofactor utilization and tropism in a dualtropic human immunodeficiency virus type 1 primary isolate. J Virol 72: 4478–4484. 9557745 10.1128/jvi.72.5.4478-4484.1998 PMC109685

[35] Cocchi F, DeVico AL, Garzino-Demo A, Cara A, Gallo RC, et al (1996) The V3 domain of the HIV-1 gp120 envelope glycoprotein is critical for chemokine-mediated blockade of infection. Nat Med 2: 1244–1247. 8898753 10.1038/nm1196-1244

[36] Nabatov AA, Pollakis G, Linnemann T, Kliphius A, Chalaby MI, et al (2004) Intrapatient alterations in the human immunodeficiency virus type 1 gp120 V1V2 and V3 regions differentially modulate coreceptor usage, virus inhibition by CC/CXC chemokines, soluble CD4, and the b12 and 2G12 monoclonal antibodies. J Virol 78: 524–530. 14671134 10.1128/JVI.78.1.524-530.2004 PMC303404

[37] Sander O, Sing T, Sommer I, Low AJ, Cheung PK, et al (2007) Structural descriptors of gp120 V3 loop for the prediction of HIV-1 coreceptor usage. PLoS Comput Biol 3: e58. 17397254 10.1371/journal.pcbi.0030058 PMC1848001

[38] Speck RF, Wehrly K, Platt EJ, Atchison RE, Charo IF, et al (1997) Selective employment of chemokine receptors as human immunodeficiency virus type 1 coreceptors determined by individual amino acids within the envelope V3 loop. J Virol 71: 7136–7139. 9261451 10.1128/jvi.71.9.7136-7139.1997 PMC192016

[39] Wu L, Gerard NP, Wyatt R, Choe H, Parolin C, et al (1996) CD4-induced interaction of primary HIV-1 gp120 glycoproteins with the chemokine receptor CCR-5. Nature 384: 179–183. 8906795 10.1038/384179a0

[40] De Jong JJ, De Ronde A, Keulen W, Tersmette M, Goudsmit J (1992) Minimal requirements for the human immunodeficiency virus type 1 V3 domain to support the syncytium-inducing phenotype: analysis by single amino acid substitution. J Virol 66: 6777–6780. 1404617 10.1128/jvi.66.11.6777-6780.1992 PMC240176

[41] Clevestig P, Pramanik L, Leitner T, Ehrnst A (2006) CCR5 use by human immunodeficiency virus type 1 is associated closely with the gp120 V3 loop N-linked glycosylation site. J Gen Virol 87: 607–612. 16476981 10.1099/vir.0.81510-0

[42] Fouchier RA, Groenink M, Kootstra NA, Tersmette M, Huisman HG, et al (1992) Phenotype-associated sequence variation in the third variable domain of the human immunodeficiency virus type 1 gp120 molecule. J Virol 66: 3183–3187. 1560543 10.1128/jvi.66.5.3183-3187.1992 PMC241084

[43] Polzer S, Dittmar MT, Schmitz H, Schreiber M (2002) The N-linked glycan g15 within the V3 loop of the HIV-1 external glycoprotein gp120 affects coreceptor usage, cellular tropism, and neutralization. Virology 304: 70–80. 12490404 10.1006/viro.2002.1760

[44] Resch W, Hoffman N, Swanstrom R (2001) Improved success of phenotype prediction of the human immunodeficiency virus type 1 from envelope variable loop 3 sequence using neural networks. Virology 288: 51–62. 11543657 10.1006/viro.2001.1087

[45] De Wolf F, Hogervorst E, Goudsmit J, Fenyo EM, Rubsamen-Waigmann H, et al (1994) Syncytium-inducing and non-syncytium-inducing capacity of human immunodeficiency virus type 1 subtypes other than B: phenotypic and genotypic characteristics. WHO Network for HIV Isolation and Characterization. AIDS Res Hum Retroviruses 10: 1387–1400. 7888192 10.1089/aid.1994.10.1387

[46] Briggs DR, Tuttle DL, Sleasman JW, Goodenow MM (2000) Envelope V3 amino acid sequence predicts HIV-1 phenotype (co-receptor usage and tropism for macrophages). AIDS 14: 2937–2939. 11153675 10.1097/00002030-200012220-00016

[47] Jensen MA, Li FS, van 't Wout AB, Nickle DC, Shriner D, et al (2003) Improved coreceptor usage prediction and genotypic monitoring of R5-to-X4 transition by motif analysis of human immunodeficiency virus type 1 env V3 loop sequences. J Virol 77: 13376–13388. 14645592 10.1128/JVI.77.24.13376-13388.2003 PMC296044

[48] Pillai S, Good B, Richman D, Corbeil J (2003) A new perspective on V3 phenotype prediction. AIDS Res Hum Retroviruses 19: 145–149. 12643277 10.1089/088922203762688658

[49] Chang VT, Crispin M, Aricescu AR, Harvey DJ, Nettleship JE, et al (2007) Glycoprotein structural genomics: solving the glycosylation problem. Structure 15: 267–273. 17355862 10.1016/j.str.2007.01.011 PMC1885966

[50] Depetris RS, Julien JP, Khayat R, Lee JH, Pejchal R, et al (2012) Partial enzymatic deglycosylation preserves the structure of cleaved recombinant HIV-1 envelope glycoprotein trimers. J Biol Chem 287: 24239–24254. 22645128 10.1074/jbc.M112.371898 PMC3397850

[51] Zhou T, Xu L, Dey B, Hessell AJ, Van Ryk, Donald, et al (2007) Structural definition of a conserved neutralization epitope on HIV-1 gp120. Nature 445: 732–737. 17301785 10.1038/nature05580 PMC2584968

[52] Huang C-c, Tang M, Zhang M-Y, Majeed S, Montabana E, et al (2005) Structure of a V3-containing HIV-1 gp120 core. Science 310: 1025–1028. 16284180 10.1126/science.1118398 PMC2408531

[53] Kwong PD, Wyatt R, Robinson J, Sweet RW, Sodroski J, et al (1998) Structure of an HIV gp120 envelope glycoprotein in complex with the CD4 receptor and a neutralizing human antibody. Nature 393: 648–659. 9641677 10.1038/31405 PMC5629912

[54] Mao Y, Wang L, Gu C, Herschhorn A, Xiang SH, et al (2012) Subunit organization of the membrane-bound HIV-1 envelope glycoprotein trimer. Nat Struct Mol Biol 19: 893–899. 22864288 10.1038/nsmb.2351 PMC3443289

[55] Kong L, Lee JH, Doores KJ, Murin CD, Julien JP, et al (2013) Supersite of immune vulnerability on the glycosylated face of HIV-1 envelope glycoprotein gp120. Nat Struct Mol Biol 20: 796–803. 23708606 10.1038/nsmb.2594 PMC3823233

[56] Durrant JD, McCammon JA (2011) Molecular dynamics simulations and drug discovery. BMC Biol 9: 71. 22035460 10.1186/1741-7007-9-71 PMC3203851

[57] Karplus M, McCammon JA (2002) Molecular dynamics simulations of biomolecules. Nat Struct Biol 9: 646–652. 12198485 10.1038/nsb0902-646

[58] Woods RJ, Tessier MB (2010) Computational glycoscience: characterizing the spatial and temporal properties of glycans and glycan-protein complexes. Curr Opin Struct Biol 20: 575–583. 20708922 10.1016/j.sbi.2010.07.005 PMC3936461

[59] Yokoyama M, Naganawa S, Yoshimura K, Matsushita S, Sato H (2012) Structural Dynamics of HIV-1 Envelope Gp120 Outer Domain with V3 Loop. PLoS ONE 7: e37530. 22624045 10.1371/journal.pone.0037530 PMC3356331

[60] Lopez de Victoria A, Tamamis P, Kieslich CA, Morikis D (2012) Insights into the structure, correlated motions, and electrostatic properties of two HIV-1 gp120 V3 loops. PLoS ONE 7: e49925. 23185486 10.1371/journal.pone.0049925 PMC3501474

[61] Pettersen EF, Goddard TD, Huang CC, Couch GS, Greenblatt DM, et al (2004) UCSF Chimera-a visualization system for exploratory research and analysis. Journal of Computational Chemistry 25: 1605–1612. 15264254 10.1002/jcc.20084

[62] Gray ES, Moore PL, Pantophlet RA, Morris L (2007) N-linked glycan modifications in gp120 of human immunodeficiency virus type 1 subtype C render partial sensitivity to 2G12 antibody neutralization. J Virol 81: 10769–10776. 17634239 10.1128/JVI.01106-07 PMC2045459

[63] Yang Q, Li C, Wei Y, Huang W, Wang L-X (2010) Expression, glycoform characterization, and antibody-binding of HIV-1 V3 glycopeptide domain fused with human IgG1-Fc. Bioconjug Chem 21: 875–883. 20369886 10.1021/bc9004238 PMC2901909

[64] Sanders RW, van Anken E, Nabatov AA, Liscaljet IM, Bontjer I, et al (2008) The carbohydrate at asparagine 386 on HIV-1 gp120 is not essential for protein folding and function but is involved in immune evasion. Retrovirology 5: 10. 18237398 10.1186/1742-4690-5-10 PMC2262092

[65] Sanders RW, Venturi M, Schiffner L, Kalyanaraman R, Katinger H, et al (2002) The mannose-dependent epitope for neutralizing antibody 2G12 on human immunodeficiency virus type 1 glycoprotein gp120. J Virol 76: 7293–7305. 12072528 10.1128/JVI.76.14.7293-7305.2002 PMC136300

[66] Raska M, Takahashi K, Czernekova L, Zachova K, Hall S, et al (2010) Glycosylation patterns of HIV-1 gp120 depend on the type of expressing cells and affect antibody recognition. J Biol Chem 285: 20860–20869. 20439465 10.1074/jbc.M109.085472 PMC2898351

[67] Losman B, Biller M, Olofsson S, Schonning K, Lund OS, et al (1999) The N-linked glycan of the V3 region of HIV-1 gp120 and CXCR4-dependent multiplication of a human immunodeficiency virus type 1 lymphocyte-tropic variant. FEBS Letters 454: 47–52. 10413093 10.1016/s0014-5793(99)00740-1

[68] Hu Q, Mahmood N, Shattock RJ (2007) High-mannose-specific deglycosylation of HIV-1 gp120 induced by resistance to cyanovirin-N and the impact on antibody neutralization. Virology 368: 145–154. 17658575 10.1016/j.virol.2007.06.029 PMC2121147

[69] Case DA, Darden TA, Cheatham TE, III, Simmerling CL, et al.. (2008) AMBER 10. University of California, San Francisco.

[70] Hornak V, Abel R, Okur A, Strockbine B, Roitberg A, et al (2006) Comparison of multiple Amber force fields and development of improved protein backbone parameters. Proteins 65: 712–725. 16981200 10.1002/prot.21123 PMC4805110

[71] Kirschner KN, Yongye AB, Tschampel SM, Gonzalez-Outeirino J, Daniels CR, et al (2008) GLYCAM06: a generalizable biomolecular force field. Carbohydrates. J Comput Chem 29: 622–655. 17849372 10.1002/jcc.20820 PMC4423547

[72] Wehle M, Vilotijevic I, Lipowsky R, Seeberger PH, Silva DV, et al (2012) Mechanical compressibility of the glycosylphosphatidylinositol (GPI) anchor backbone governed by independent glycosidic linkages. J Am Chem Soc 134: 18964–18972. 23061547 10.1021/ja302803r

[73] DePaul AJ, Thompson EJ, Patel SS, Haldeman K, Sorin EJ (2010) Equilibrium conformational dynamics in an RNA tetraloop from massively parallel molecular dynamics. Nucleic Acids Res 38: 4856–4867. 20223768 10.1093/nar/gkq134 PMC2919701

[74] Sorin EJ, Pande VS (2005) Exploring the helix-coil transition via all-atom equilibrium ensemble simulations. Biophys J 88: 2472–2493. 15665128 10.1529/biophysj.104.051938 PMC1305346

[75] Berendsen H vdSD, Drunen R (1995) GROMACS: a message-passing parallel molecular dynamics implementation. Comp Phys Comm 91: 43–56.

[76] Hess B KC, van der Spoel D, Lindahl E (2008) GROMACS 4: algorithms for highly efficient, load-balanced, and scalable molecular simulations. J Chem Theory Comput 4: 435–447. 26620784 10.1021/ct700301q

[77] Jorgensen WL, Chandrasekhar J, Madura JD, Impey RW, Klein ML (1983) Comparison of simple potential functions for simulating liquid water. The Journal of Chemical Physics 79: 926–935.

[78] Essmann U, Perera L, Berkowitz ML, Darden T, Lee H, et al (1995) A smooth particle mesh Ewald method. The Journal of Chemical Physics 103: 8577–8593.

[79] Westby M, van der Ryst E (2010) CCR5 antagonists: host-targeted antiviral agents for the treatment of HIV infection, 4 years on. Antivir Chem Chemother 20: 179–192. 20413825 10.3851/IMP1507

[80] R_Core_Team (2012) R: A Language and Environment for Statistical Computing. R Foundation for Statistical Computing. Vienna, Austria.

[81] Humphrey W, Dalke A, Schulten K (1996) VMD: visual molecular dynamics. J Mol Graph 14: 33–38, 27–38. 10.1016/0263-7855(96)00018-5 8744570

[82] Joao HC, Dwek RA (1993) Effects of glycosylation on protein structure and dynamics in ribonuclease B and some of its individual glycoforms. Eur J Biochem 218: 239–244. 8243469 10.1111/j.1432-1033.1993.tb18370.x

[83] Zheng K, Bantog C, Bayer R (2011) The impact of glycosylation on monoclonal antibody conformation and stability. MAbs 3: 568–576. 22123061 10.4161/mabs.3.6.17922 PMC3242843

[84] Wang X, Kumar S, Buck PM, Singh SK (2013) Impact of deglycosylation and thermal stress on conformational stability of a full length murine IgG2a monoclonal antibody: observations from molecular dynamics simulations. Proteins 81: 443–460. 23065923 10.1002/prot.24202

[85] Liu J, Bartesaghi A, Borgnia MJ, Sapiro G, Subramaniam S (2008) Molecular architecture of native HIV-1 gp120 trimers. Nature 455: 109–113. 18668044 10.1038/nature07159 PMC2610422

[86] Sattentau QJ, Moore JP (1991) Conformational changes induced in the human immunodeficiency virus envelope glycoprotein by soluble CD4 binding. J Exp Med 174: 407–415. 1713252 10.1084/jem.174.2.407 PMC2118908

[87] Sing T, Low AJ, Beerenwinkel N, Sander O, Cheung PK, et al (2007) Predicting HIV coreceptor usage on the basis of genetic and clinical covariates. Antivir Ther 12: 1097–1106. 18018768

[88] Xu S, Huang X, Xu H, Zhang C (2007) Improved prediction of coreceptor usage and phenotype of HIV-1 based on combined features of V3 loop sequence using random forest. J Microbiol 45: 441–446. 17978804

[89] Kwong PD, Doyle ML, Casper DJ, Cicala C, Leavitt SA, et al (2002) HIV-1 evades antibody-mediated neutralization through conformational masking of receptor-binding sites. Nature 420: 678–682. 12478295 10.1038/nature01188

[90] Myszka DG, Sweet RW, Hensley P, Brigham-Burke M, Kwong PD, et al (2000) Energetics of the HIV gp120-CD4 binding reaction. Proc Natl Acad Sci U S A 97: 9026–9031. 10922058 10.1073/pnas.97.16.9026 PMC16815

[91] Benson NC, Daggett V (2008) Dynameomics: large-scale assessment of native protein flexibility. Protein Sci 17: 2038–2050. 18796694 10.1110/ps.037473.108 PMC2590920

[92] Carpenter EP, Beis K, Cameron AD, Iwata S (2008) Overcoming the challenges of membrane protein crystallography. Curr Opin Struct Biol 18: 581–586. 18674618 10.1016/j.sbi.2008.07.001 PMC2580798

[93] Kuroda D, Shirai H, Jacobson MP, Nakamura H (2012) Computer-aided antibody design. Protein Eng Des Sel 25: 507–521. 22661385 10.1093/protein/gzs024 PMC3449398

[94] Lindorff-Larsen K, Piana S, Palmo K, Maragakis P, Klepeis JL, et al (2010) Improved side-chain torsion potentials for the Amber ff99SB protein force field. Proteins 78: 1950–1958. 20408171 10.1002/prot.22711 PMC2970904

[95] Dror RO, Dirks RM, Grossman JP, Xu H, Shaw DE (2012) Biomolecular simulation: a computational microscope for molecular biology. Annu Rev Biophys 41: 429–452. 22577825 10.1146/annurev-biophys-042910-155245

[96] Tan Q, Zhu Y, Li J, Chen Z, Han GW, et al (2013) Structure of the CCR5 chemokine receptor-HIV entry inhibitor maraviroc complex. Science 341: 1387–1390. 24030490 10.1126/science.1241475 PMC3819204

[97] Pikora C, Wittish C, Desrosiers RC (2005) Identification of two N-linked glycosylation sites within the core of the simian immunodeficiency virus glycoprotein whose removal enhances sensitivity to soluble CD4. J Virol 79: 12575–12583. 16160185 10.1128/JVI.79.19.12575-12583.2005 PMC1211561

