# Supplementary figures and images for: The Influence of N-Linked Glycans on the Molecular Dynamics of the HIV-1 gp120 V3 Loop
Source: PLoS One. 2013 Nov 26;8(11):e80301. doi: 10.1371/journal.pone.0080301 (PMC3841175; doi:10.1371/journal.pone.0080301)

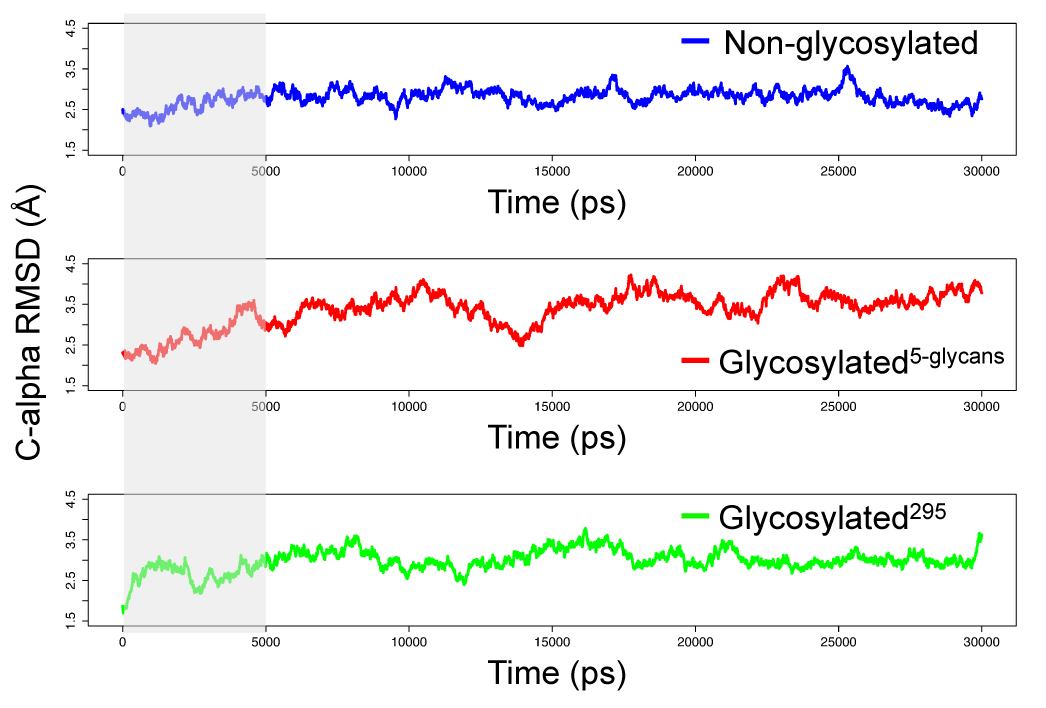

Supplement: Figure S1 — RMSF values for the C-αlpha atoms. These values represent the entire gp120 for the non-glycosylated, glycosylated5-glycans, and glycosylated295 trajectories. The shaded areas represent the part of the trajectory that was discarded as burn-in. (TIF) [file pone.0080301.s001.tif]

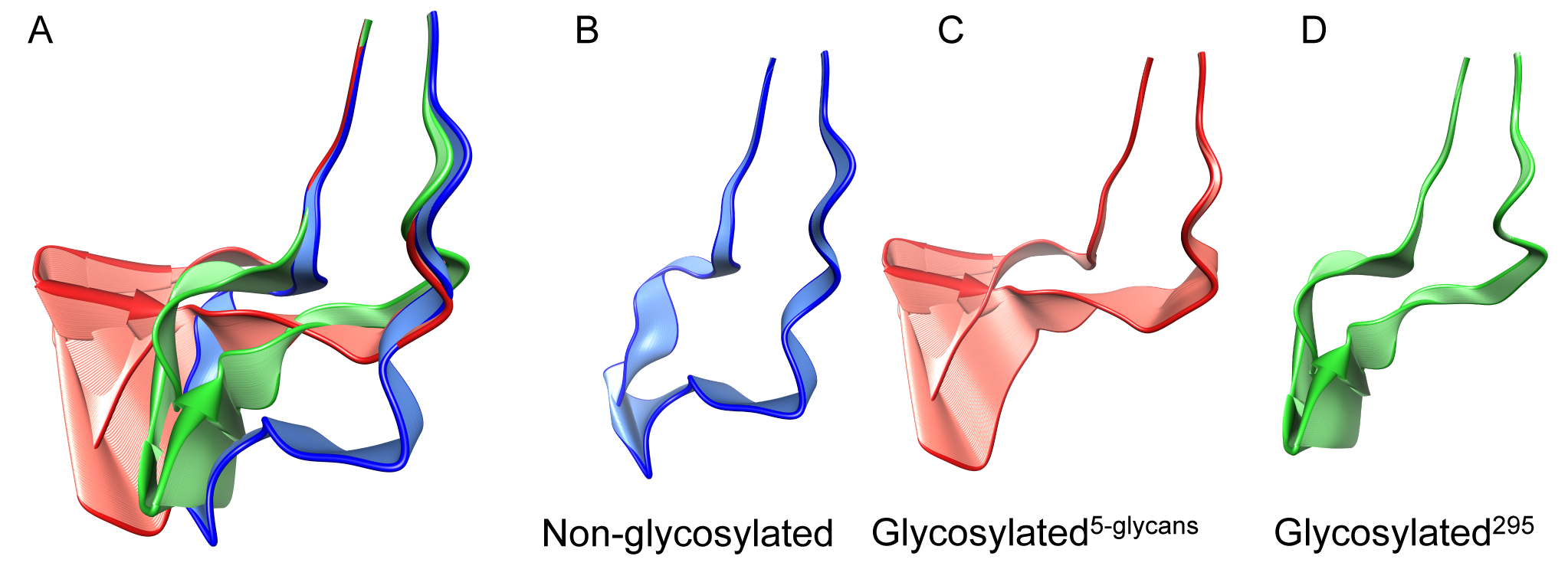

Supplement: Figure S2 — Representation of the range of movement of the V3 loops for the second principal component. The range of movement is presented for (A) all systems, (B) non-glycosylated, (C) glycosylated5-glycans, and (D) glycosylated295 trajectories. The shaded colors represent the intermediate positions between the extremes. (TIF) [file pone.0080301.s002.tif]

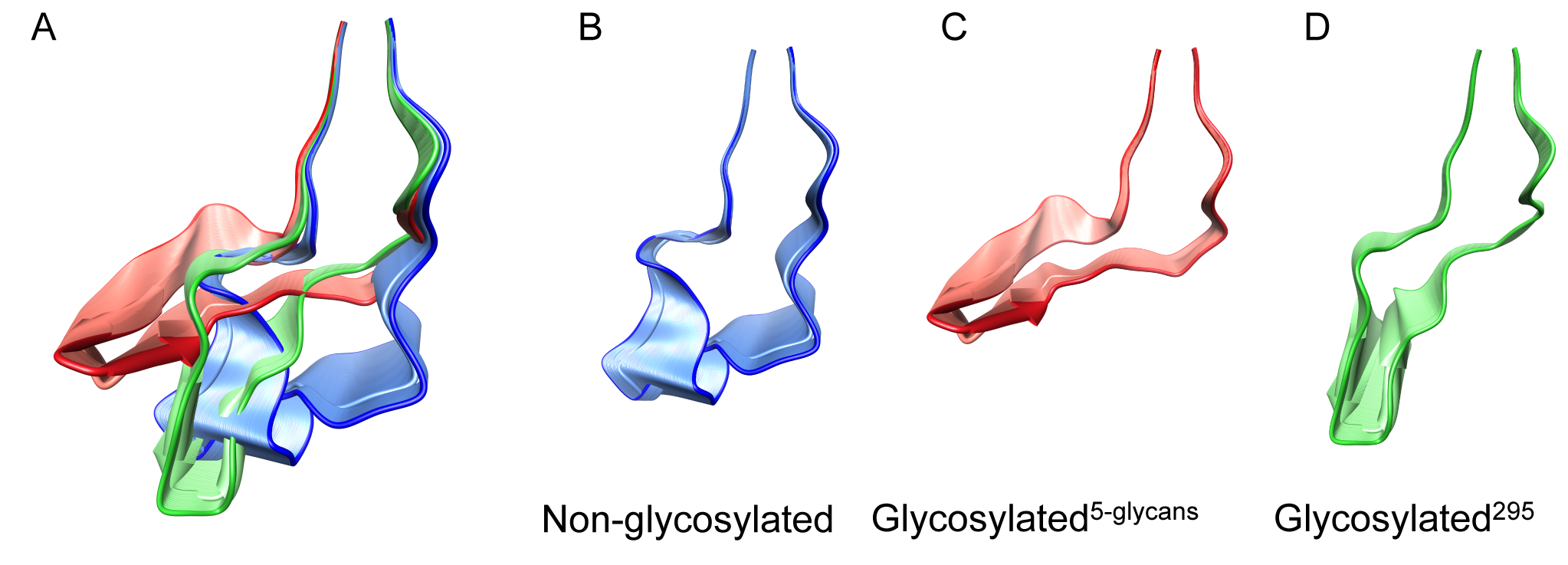

Supplement: Figure S3 — Representation of the range of movement of the V3 loops for the third principal component. The range of movement is presented for (A) all systems, (B) non-glycosylated, (C) glycosylated5-glycans, and (D) glycosylated295 trajectories. The shaded colors represent the intermediate positions between the extremes. (TIF) [file pone.0080301.s003.tif]

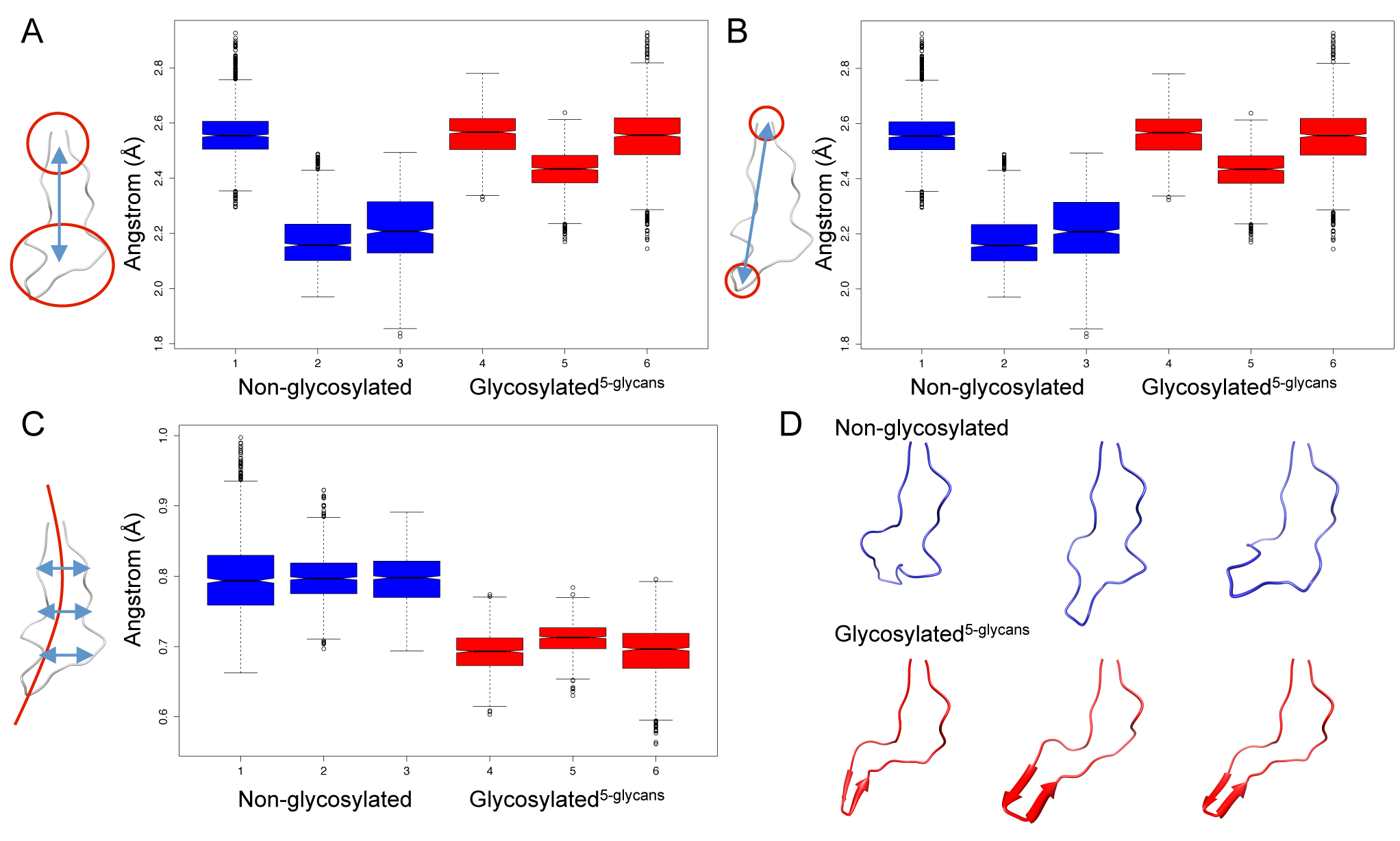

Supplement: Figure S4 — Comparison between distance distributions for regions of the V3 loop. Comparison between the distance distributions for the centers of mass between the base and tip of the V3 loop (A and B) and between the two sides of the V3 loop (C); and the corresponding average V3 loop structures (D) using the entire 10 ns of the uncorrelated trajectories for the three non-glycosylated (blue) and three glycosylated5-glycans­ (red) additional examples. (TIF) [file pone.0080301.s004.tif]

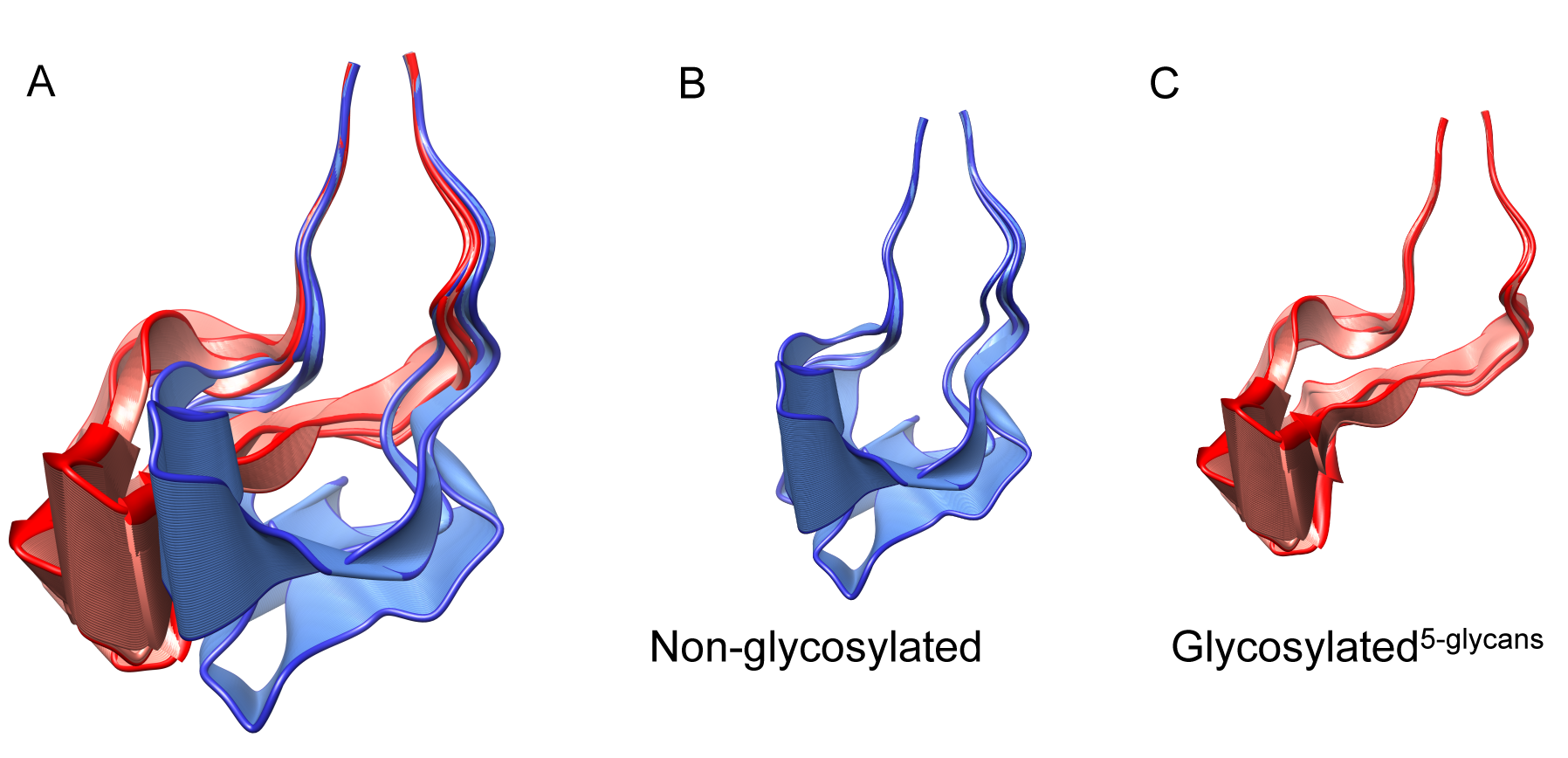

Supplement: Figure S5 — Representation of the range of movement of the V3 loops for the first principal component. The range of movement is presented for (A) non-glycosylated and glycosylated5-glycans, (B) non-glycosylated, and (C) glycosylated5-glycans trajectories. The shaded colors represent the intermediate positions between the extremes. (TIF) [file pone.0080301.s005.tif]

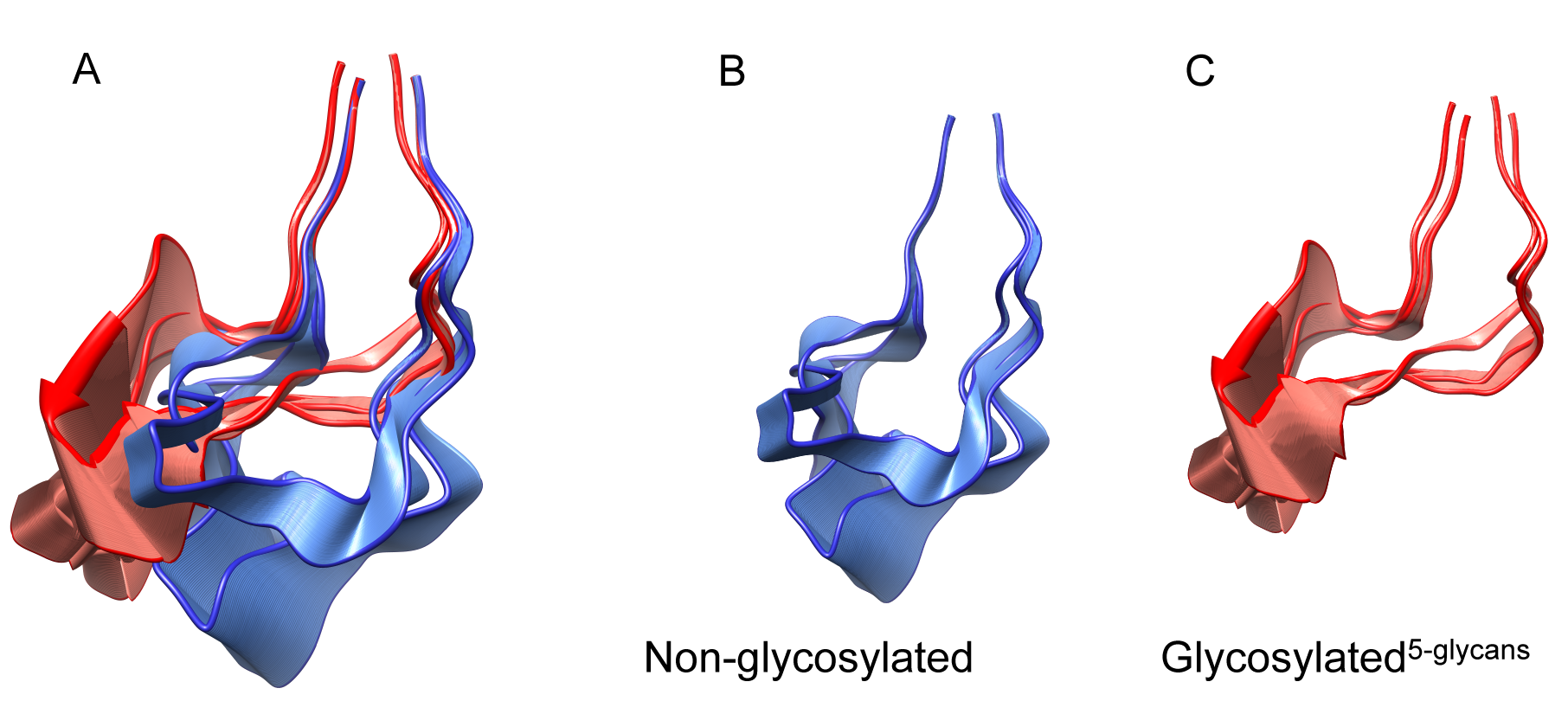

Supplement: Figure S6 — Representation of the range of movement of the V3 loops for the second principal component. The range of movement is presented for (A) non-glycosylated and glycosylated5-glycans, (B) non-glycosylated, and (C) glycosylated5-glycans trajectories. The shaded colors represent the intermediate positions between the extremes. (TIF) [file pone.0080301.s006.tif]

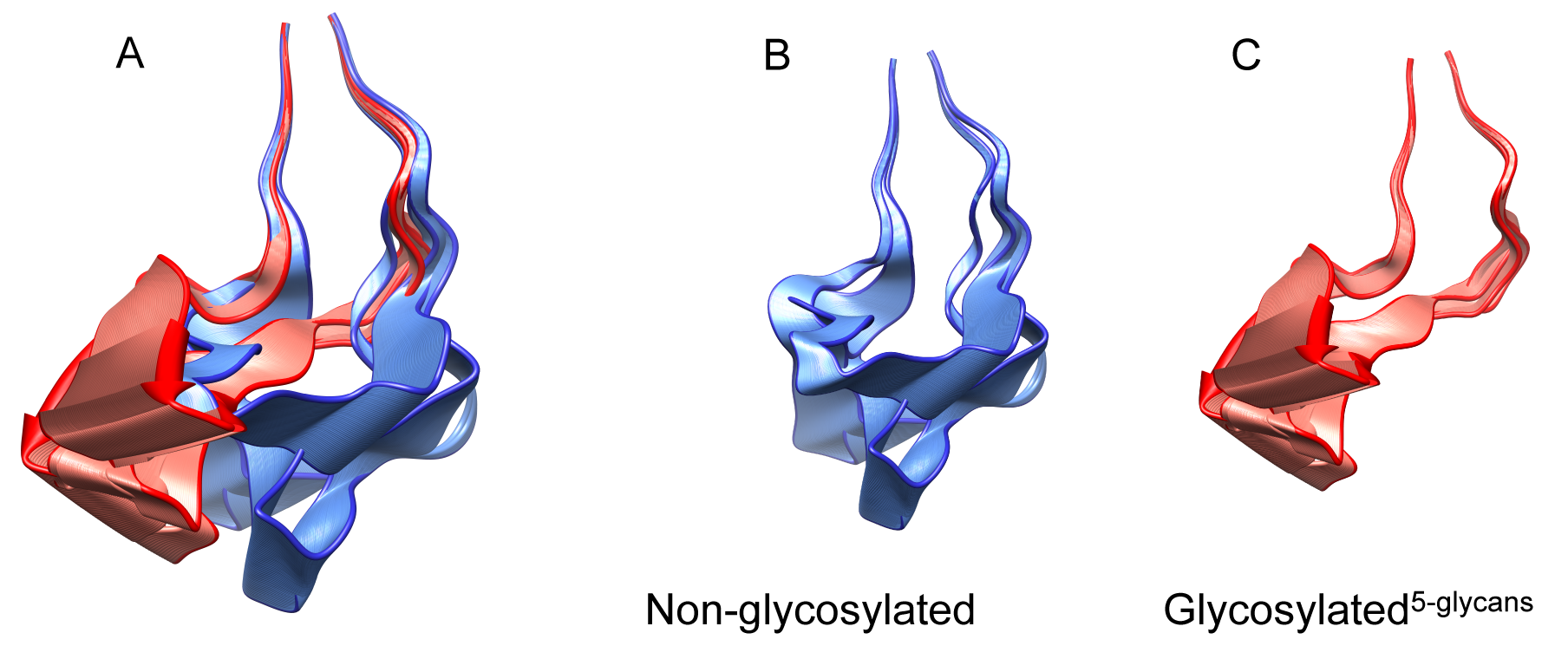

Supplement: Figure S7 — Representation of the range of movement of the V3 loops for the third principal component. The range of movement is presented for (A) non-glycosylated and glycosylated5-glycans, (B) non-glycosylated, and (C) glycosylated5-glycans trajectories. The shaded colors represent the intermediate positions between the extremes. (TIF) [file pone.0080301.s007.tif]
