# Supplementary material for: The Influence of N-Linked Glycans on the Molecular Dynamics of the HIV-1 gp120 V3 Loop
Source: PLoS One. 2013 Nov 26;8(11):e80301. doi: 10.1371/journal.pone.0080301 (PMC3841175; doi:10.1371/journal.pone.0080301)
Supplement: Table S1 — Percentage motion included in each of the first three, and the total sum of the first three, principal components (PCs) for each of the uncorrelated repeats (10 ns). (PDF) [file pone.0080301.s008.pdf]

	PC1 (%)	PC2 (%)	PC3 (%)	Total (%)
**1 Non-glycosylated**	30	28	10	68
**2 Non-glycosylated**	57	19	5	81
**3 Non-glycosylated**	38	19	8	65
**1 Glycosylated**$^{5\text{-glycans}}$	48	12	9	69
**2 Glycosylated**$^{5\text{-glycans}}$	47	24	4	75
**3 Glycosylated**$^{5\text{-glycans}}$	50	14	10	74
